# Computerized Cognitive–Behavioral Therapy

**DOI:** 10.35946/arcr.v36.1.13

**Published:** 2014

**Authors:** Kathleen M. Carroll

**Affiliations:** Kathleen M. Carroll, Ph.D., is the Albert E. Kent Professor of Psychiatry in the Department of Psychiatry at Yale University School of Medicine, New Haven, Connecticut.

With an estimated 90 percent or more of alcohol use disorders going untreated (Substance Abuse and Mental Health Services Administration 2012), the search for interventions that easily, effectively, and economically reach more people has become a priority. The landmark 1990 report, *Broadening the Base of Treatment for Alcohol Problems* (Institute of Medicine 1990), refocused alcohol treatment research toward an emphasis on developing, standardizing, and disseminating new behavioral therapies to expand the reach of alcohol treatment. A particularly exciting development on this front has been the creation of computerized versions of interventions shown to be effective in clinical settings.

Computerized treatments have multiple potential advantages for expanding the base of treatment for alcohol use disorders, including broad availability 24 hours a day, lower cost, standardization, greater ability to reach rural and underserved populations, and greater confidentiality, leading to fewer concerns about stigma (Carroll and Rounsaville 2010; Cunningham and Van Mierlo 2009). In effect, computer-based interventions can serve as “clinician extenders,” offering a means of delivering high-quality, standardized versions of screening, evaluation, and brief treatments, at relatively low cost. That said, these interventions are relatively new, and, therefore, both their quality and the level of rigor of the studies supporting them varies widely (Carey et al. 2009; Kiluk et al. 2011; Rooke et al. 2010). Here, we will highlight only approaches with at least preliminary validation in clinical trials.

## Electronic Screenings and Brief Interventions (eSBIs)

Many Web sites exist that allow people to assess their alcohol use from their personal computers or other devices using Web-based versions of more traditional, clinician-delivered SBIs (Babor et al. 2007). These sites connect people with SBI services immediately, when their motivation may be highest, rather than asking them to wait several days or weeks for an appointment with a clinician. Called electronic SBI (eSBI), these sites typically are based on principles of clinician-delivered SBIs, using a validated instrument such as the Alcohol Use Disorders Identification Test (AUDIT) to assess alcohol use and risk (Allen et al. 1997; Bohn et al. 1995), provide feedback about the user’s level of risk, and offer some suggestions or additional resources for reducing drinking.

Many eSBIs exist; however, only a few have been evaluated in randomized clinical trials, and the majority of those studies have been conducted on college populations and may not generalize to broader society (Bewick et al. 2008; Rooke et al. 2010; White et al. 2010). In fact, one recent metaanalysis found only 17 randomized controlled trials of eSBIs that provided enough data for comparison, and 13 of those studied student populations (Donoghue et al. 2014). Despite this limitation, Donoghue and colleagues reported that the eSBIs studied had a significant effect on participants’ drinking behavior for up to 12 months postintervention. Overall, studies of eSBIs find a small but significant effect size for eSBIs and conclude that some users can benefit from these computer-based interventions, particularly people unlikely to seek out more traditional services (Bewick et al. 2008; Donoghue et al. 2014; Rooke et al. 2010; White et al. 2010).

To date, the English-language eSBIs designed for the general public that have the strongest evidence supporting their efficacy based on randomized controlled trials are The Drinker’s Checkup (www.drinkerscheckup.com) (Hester et al. 2005) and Check Your Drinking (www.checkyourdrinking.net) (Cunningham et al. 2009).

## Web-Based Multisession Interventions

Compared with eSBIs, fewer computer-based intensive, multiple-session interventions for alcohol use disorders exist, and even fewer have been tested with randomized controlled clinical trials. Those have shown some promise.

One study, for example, examined whether study participants who utilized the Check Your Drinking SBI would get an added benefit if also offered an extended Internet intervention called the Alcohol Help Center (AHC). AHC provides cognitive–behavioral, motivational, and relapse prevention components that previous research has shown helps problem drinkers (Cunningham 2012). People using the AHC can complete whichever exercises they choose in whatever order they like over an unspecified, extended period of time. The study recruited 170 problem drinkers from the general population and randomly assigned them access to Check Your Drinking alone or Check Your Drinking along with AHC. Ninety percent of participants returned a 6-month follow-up questionnaire that assessed their drinking behavior. Both groups significantly reduced their drinking, but participants who accessed AHC showed an added benefit of the extended intervention. The study did not assess how often study participants engaged with AHC.

Another study of nondependent problem drinkers showed that online training in moderation management using the “Moderate Drinking” application (www.moderatedrinking.com) combined with online moderation management through the Moderation Management Web site (www.moderation.org) is effective in reducing drinking days (Hester et al. 2011). The study randomly assigned 78 participants to either use the two interventions in tandem or to just use Moderation Management. Although both groups significantly decreased the amount they drank, even after a full year, participants that used both Web sites had a higher percentage of days abstinent and fewer alcohol-related problems than the group utilizing Moderation Management only. This study did not report participants’ level of engagement with the interventions.

A more structured, 6-week online cognitive–behavioral self-help intervention for adult problem drinkers also showed promise in a randomized controlled trial conducted in the Netherlands (Riper et al. 2008). Participants who utilized the interactive self-help intervention reduced their drinking significantly more than participants who received an online psychoeducational brochure about alcohol use. Specifically, 17 percent of those receiving the intervention reduced their drinking to levels considered low risk in the Netherlands (no more than two units or 20 g of alcohol per day) compared with 5.4 percent of those receiving the brochure. Overall, the intervention group decreased their weekly alcohol consumption significantly more than the control group.

Although those findings are promising, another study of adult problem drinkers in the Netherlands suggests that it might be more effective to combine online self-help interventions with Internet-based one-on-one therapy (Blankers and Koeter 2011). The randomized controlled trial assigned 205 problem drinkers to one of three interventions:

- A waitlist for treatment (the no treatment control);
- Self-Help Alcohol Online (SAO), a fully automated, Internet based, self-guided treatment program based on a cognitive–behavioral treatment (CBT)/motivational interviewing (MI) treatment protocol; or
- Therapy Alcohol Online (TAO), which provides the same CBT/MI treatment protocol as SAO but also includes up to seven synchronous text-based chat-therapy sessions with a trained therapist.

Three months after starting the program, study participants in both treatment groups had reduced their alcohol consumption and their level of alcohol-related problems significantly more than those on the waitlist, but there was no significant difference between the treatment groups. That changed after 6 months when participants receiving TAO showed larger reductions in alcohol consumption than those receiving SAO. The researchers concluded that both TAO and SAO effectively reduced drinking and drinking-related problems but that TAO seemed to lead to better results after 6 months.

A recent meta-analysis comparing nine randomized controlled clinical trials of guided and unguided low-intensity Internet interventions for adults (the authors excluded studies of college students) found that Internet interventions had a small but significant effect on drinking behavior (Riper et al. 2014). Participants in the Internet interventions drank an average of 22 grams per week less than participants in control groups and were more likely to adhere to low-risk drinking guidelines postintervention. Riper and colleagues note that, although the effect sizes of these interventions are small (*g* = 0.20), because they have the potential to reach so many people, they could have a large influence on public health.

A higher-intensity computer-based intervention that shows promise is computer-based training for CBT (CBT4CBT). This eight-session computer-based version of CBT focuses on teaching basic coping skills, presenting video examples of effective coping skills used in a number of realistic situations and providing opportunities for patients to practice and review new skills. Two completed trials indicate that CBT4CBT improves outcomes over standard treatment alone. One study (Carroll 2008) tested CBT4CBT in an outpatient setting with a mixed group of 77 substance users, including a large number of alcohol-dependent individuals. The other study tested the intervention among 101 cocaine-dependent methadone-maintained patients (Carroll et al. 2014). Both studies found CBT4CBT had a durable effect on substance use, with improvement in substance use increasing over time, suggesting that CBT’s “sleeper effect” is retained in its Web-based version (Carroll et al. 2009). These studies also found that CBT4CBT effectively taught the targeted skills and that skill acquisition in turn mediated the effects on substance use (Kiluk et al. 2010). Researchers recently have developed a version of CBT4CBT specifically for individuals with alcohol use disorders and have begun randomized clinical trials evaluating its efficacy, including one evaluating CBT4CBT as a standalone intervention. More information can be found at the Web site: www.cbt4cbt.com.

## Conclusion

Computer and Web-based interventions hold great promise for reaching the large number of individuals who may benefit from alcohol treatment but do not access it. Thus far, the meta-analytic work in this area points to a modest but significant effect of these interventions and hence their potential to improve public health by extending the reach of interventions beyond the clinic.

At the same time, enthusiasm regarding the potential of these interventions should be tempered with some caution. It is critical to carefully evaluate these interventions before they are broadly disseminated. Relatively few of the many available Web-based interventions have been carefully evaluated in well-controlled clinical trials (Kiluk et al. 2011), and the conclusions that can be drawn from many studies are constrained by high levels of dropout, high attrition, and weak control conditions (e.g., waitlists). Indeed, recent meta-analyses have included only one-tenth of available published reports (Riper et al. 2014) because of methodological limitations. The field, while not still in its infancy, remains young, and basic questions regarding which individuals are best served by and most responsive to online versus face-to-face interventions have not been addressed (Carey et al. 2012). That said, if research demonstrates computer-based interventions to be safe and even moderately effective, they may have tremendous impact for individuals with alcohol use disorders and their families, potentially reaching people who would not access more traditional treatment options.
